# Concept Creep and Psychiatrization

**DOI:** 10.3389/fsoc.2021.806147

**Published:** 2021-12-16

**Authors:** Nick Haslam, Jesse S. Y. Tse, Simon De Deyne

**Affiliations:** School of Psychological Sciences, University of Melbourne, Parkville, VIC, Australia

**Keywords:** concept creep, diagnosis, harm, psychiatric classification, over-diagnosis

## Abstract

Some aspects of psychiatrization can be understood as forms of concept creep, the progressive expansion of concepts of harm. This article compares the two concepts and explores how concept creep sheds light on psychiatrization. We argue that although psychiatrization is in some respects a broader concept than concept creep, addressing institutional and societal dimensions of the expanding reach of psychiatry in addition to conceptual change, concept creep is broader in other respects, viewing the expansion of psychiatric concepts as examples of the broadening of a more extensive range of harm-related concepts. A concept creep perspective on psychiatrization clarifies the different forms of expansion it involves, the centrality of harm to it, its benefits as well as its costs, its variations across individuals and groups, and the drivers of psychiatrization in the general public and in fields beyond psychiatry.

## Introduction

The concept of psychiatrization identifies a pattern of correlated societal and cultural changes that have been underway for several decades but now seem especially urgent to address. Beeker et al. s (2021) review of the field points to rising rates of mental illness, increasing mental health service utilization, and evidence of over-diagnosis, over-treatment, and over-prescription (Paris, 2020). Coupled with these changes is an expansion in the number and inclusiveness of psychiatric diagnoses that has led critics to lead campaigns to save normality from the relentless encroachment of diagnosable pathology (Frances, 2013). Beyond changes such as these in the field of mental health, expanded understandings of mental disorder have spread in the culture at large, accompanied by popular adoption of a psychiatric idiom to make sense of everyday experiences of deviance and distress. Psychiatrization is a multi-faceted phenomenon and drawing its aspects together under the term provides researchers and theorists with an opportunity to join forces to understand it better.

In that spirit, we argue that “concept creep” offers a useful vantage-point for understanding some aspects of psychiatrization. Although that notion emerges from a different intellectual context–a psychological frame of references and an emphasis on conceptual change and its cultural dimensions–concept creep has a strong alignment with psychiatrization. Both refer to the expansion of a set of concepts and practices that has taken place over a period of decades. Both emphasize how these expansions have broad but ambivalent ramifications throughout society. In addition, some work on concept creep has explored psychiatric concepts such as mental illness and trauma.

In this article we offer an overview of theory and research on concept creep and examine how it might illuminate psychiatrization. We make no attempt to reduce one concept to the other but explore their intersections and speculate on how concept creep might deepen or challenge our understanding of some elements of psychiatrization. We conclude the article with a few specific points where the concept creep perspective might advance the study of psychiatrization.

## Concept Creep

First described by Haslam (2016), concept creep refers to the gradual expansion of the meaning of harm-related concepts. Haslam argued that several prominent psychological concepts had undergone a process of semantic inflation whereby they had come to refer to an increasingly wide range of phenomena. That broadening occurs in two directions, he argued. Concepts creep horizontally by coming to refer to qualitatively new phenomena, and vertically by coming to refer to quantitatively less extreme phenomena.

In the original paper, for example, Haslam (2016) presented case studies of six creeping concepts: abuse, addiction, bullying, mental disorder, prejudice, and trauma. The concept of bullying, for example, was initially used in the 1970s to refer to aggressive behavior among children that was intentional, repeated, and perpetrated downward in a power hierarchy. Over time, bullying expanded horizontally to include the behavior of adults in workplaces, exclusionary rather than intimidating behavior (e.g., shunning), and intimidation carried out online rather than only in person (“cyber-bullying”). Bullying also expanded vertically to include less extreme behavior such as acts that were unintentional, unrepeated, and directed at people of equal or higher power than the perpetrator.

Haslam (2016) presented similar evidence of the outward and downward spread of the other creeping concepts. He also speculated on concept creep’s causes and effects. He argued that broadened concepts of harm might result from a rising cultural preoccupation with vulnerability and risk, as proposed by Furedi (2004), and with what Pinker (2011) referred to as the “civilizing offensive” of the 1960s rights revolutions. Expansive concepts of harm problematize previously tolerated behavior and reflect a growing sensitivity to suffering and injustice. Haslam also raised the possibility that the objective decline in rates of violence and adversity in the West, also noted by Pinker, may have contributed to less severe threats being encompassed within existing concepts of harm that had previously applied only to more extreme phenomena. As levels of violent bigotry declined, for example, concepts of racism began to include subtler forms of “modern”, “aversive”, and “implicit” prejudice. In view of the centrality of harm concepts to morality, especially for social progressives, and the generally progressive thrust of most examples of concept creep, Haslam argued that it reflected in part a liberal moral agenda.

Haslam (2016) argued that the consequences of concept creep were likely to be ambivalent. On the one hand, broadened concepts of harm recognize forms of suffering and maltreatment that had previously gone unrecognized, thereby identifying them as requiring remedy and giving moral legitimacy to condemnations of harmful behavior. Defining nonviolent but negligent parenting as abuse recognizes it as a problem, just as defining significant gambling problems as addictions acknowledges their seriousness and enables new kinds of intervention. On the other hand, it can be argued that broadened concepts of harm may engender over-sensitivity to minor harms, trivialization of more severe harms, constraints on expression, and, following the theory of moral typecasting (Gray and Wegner, 2009), a polarized view of a world populated by victims and villains. Concept creep might serve some progressive political and social goals but also undermine others.

## Research on Concept Creep

Since the initial description of concept creep, a body of empirical research on the subject has emerged (see Haslam et al., 2020, for a review). Some of this work has explored its historical dimensions whereas other studies have examined the breadth of harm-related concepts as a cross-sectional analogue of concept creep. The historical research has examined several large text corpora for evidence of shifts in the prominence and meaning of harm-related concepts or presented detailed conceptual analyses of specific concepts. For example, Wheeler et al (2019) examined moral language in the Google Books corpus from 1900 to 2007 and found that harm-related morality was unique among five moral foundations (Graham et al., 2011) in demonstrating a steep rise in prominence from around 1980, consistent with a cultural account of the drivers of concept creep in recent decades.

Related work has documented the rising prominence and expanding meanings of harm-related concepts in academic discourse. Examining a corpus of about 800,000 psychology article abstracts from 1970 to 2018, Vylomova, et al. (2019) found a rise in the relative frequency of ‘addiction’, ‘bullying’, ‘harassment’, ‘prejudice’, and ‘trauma’ over the study period. Using computational linguistic methods for determining concept breadth they revealed broadening of some concepts across decades and documented specific semantic shifts (e.g., the declining association of ‘addiction’ with substances and its rising association with behaviors such as gaming). Vylomova and Haslam, (2021) extended this work by evaluating both prominence and semantic breadth of an overlapping set of creeping concepts (‘addiction’, ‘bullying’, ‘empathy’, ‘racism’, and ‘trauma’) in a general text corpus, and by exploring causal relationships between salience and breadth and between corpora. In addition to these quantitative studies, historical studies of the broadening of ‘hate’ (Haslam and Murphy, 2020) and ‘trauma’ (Haslam and McGrath, 2020) have also appeared.

Studies of individual differences in the breadth of harm-related concepts do not address concept creep directly, as they examine psychological rather than temporal variability. Nevertheless, they provide clues to the factors that might influence and be influenced by concept creep as a historical phenomenon. McGrath et al. (2019) and McGrath and Haslam (2020) demonstrated that these individual differences generalize across multiple concepts, such that people who hold relatively inclusive definitions of bullying also tended to have inclusive definitions of trauma and prejudice, for example. This finding accords with Haslam. (2016) claim that harm is the common ingredient in concept creep. These studies also pointed to a variety of demographic, personality, and ideological factors that correlate with ‘harm concept breadth’. Holding broad harm concepts is associated with being female, politically liberal, empathic, concerned about injustice toward others (but not preoccupied with injustice towards the self), likely to endorse harm-based morality, and high Neuroticism, a trait involving vulnerability to negative emotional states. Contrary to the narrative of hypersensitivity among young people, age was not associated with concept breadth. Beyond these correlates, concept breadth has been shown to predict some social judgments. In particular, people holding broader concepts of sexism and sexual harassment were more likely to judge the female victim of workplace sexism as harmed by it and deserving of compensation, and to judge the male perpetrators as more deliberate and more deserving of punishment (Chan and Haslam, 2019).

Most concept creep research has taken a wide-angle perspective on the domain of harm concepts rather than focused on the psychiatric domain. However, studies of shifts in the expansiveness of mental illness-related concepts have demonstrated the same inflationary pattern as other harm concepts. Corpus linguistic studies have revealed rises in the relative frequency (salience) and semantic breadth of trauma and addiction both in academic discourse (Vylomova et al., 2019; Vylomova and Haslam., 2021) and, somewhat less strikingly, in a general American text corpus (Haslam et al., 2016). The one notable exception to these trends for psychiatric concepts to amplify and broaden was a study not of word meanings but of shifts in the official diagnostic criteria for specific mental disorders from DSM-III to DSM-5 (Fabiano and Haslam, 2020). Contrary to expectations of a wholesale tendency for criteria to loosen, resulting in more people meeting diagnostic thresholds in more recent DSM editions, this meta-analysis of studies in which the same people were diagnosed using successive editions found no generalized pattern of diagnostic inflation. Although this finding contrasted with earlier analyses of DSM criterion sets (Boysen, 2011; Boysen and Ebersole, 2014), and with popular critiques of runaway pathologizing (Frances, 2013), it did establish strong evidence that specific disorders have inflated over time. Attention deficit hyperactivity disorder, autism, and some eating and substance use disorders exemplified this expansion.

Studies of individual differences in the breadth of mental illness concepts have also mirrored findings on individual differences in other, superficially unrelated concepts. McGrath and Haslam (2020) work revealed that people who hold more inclusive concepts of mental disorder than their peers also tend to hold more inclusive understandings of bullying and prejudice, for example, a correlation that cannot be explained by a tendency to hold more inclusive concepts of all concepts. By implication, the breadth of laypeople’s concepts of mental illness is shaped in part by their sensitivity to harm, rather than being uniquely tuned to the psychiatric domain.

Recent work by Tse and Haslam (2021) indicates that the breadth of these lay concepts has implications for mental health help-seeking. They showed that people who held more inclusive concepts of mental illness, categorizing a wider variety of experiences and actions as disordered, had more favorable help-seeking attitudes. Asian American study participants tended to hold narrower disorder concepts than White Americans, and that difference partially accounted for their less favorable attitudes, a well-established finding in cultural psychiatry. More inclusive concepts of mental illness encourage and enable people to identify an experience or behavior as a problem requiring professional attention, whereas individuals and groups with narrower concepts may be more likely to regard help-seeking as unusual and unwarranted. Whether holding more favorable views of help-seeking is interpreted positively as overcoming barriers to care or negatively as encouraging overdiagnosis and overtreatment is moot.

## Concept Creep and Psychiatrization

Concept creep theory proposes a broad historical shift in the inclusiveness of harm-related concepts that is connected to incompletely understood cultural, societal, and political changes and likely to have an array of social and psychological implications. As we have shown, empirical research is beginning to document and explore some of these processes. Psychiatrization also represents an expansionary historical trend that is likely to have complex causes and consequences. How might these two concepts be aligned? We suggest that concept creep is both narrower and broader than psychiatrization and offers a productive way to think through some of its aspects. Some of the main contrasts between the two concepts are summarized in Table 1.

**TABLE 1:** Selected contrasts been the concepts of psychiatrization and concept creep.

	Psychiatrization	Concept creep
Primary focus	Expanding reach of psychiatric institutions, practices and concepts	Semantic inflation of harm-related concepts
Disciplinary home	Medical sociology	Psychology
Explanatory emphasis	Institutional influences	Cultural influences
Domain of relevance	Psychiatry and mental illness	Concepts of harm, including psychiatric concepts

Psychiatrization is broader than concept creep in several respects. First, according to Beeker and others’ (2021) framework, psychiatrization involves not only the expansion of diagnostic categories and the broader process of pathologization–the central preoccupations of concept creep as it applies to psychiatric concepts–but also increases in the prevalence of psychiatric conditions and levels of service utilization. These increases might be understood as downstream consequences of category inflation from the standpoint of concept creep, but they are fundamental aspects of psychiatrization rather than merely effects of a more basic cause.

In addition, work on psychiatrization presents a more explicit account of the societal and institutional factors that drive the process, including the professions, the pharmaceutical and insurance industries, consumer organizations, and political forces. Writings on psychiatrization emphasize how these macro-level influences bear on the concrete realities of clinical practice and diagnostic revision. It is the combination of institutional, practice-related and conceptual shifts that is their primary focus, in accordance with psychiatrization’s disciplinary home being in medical sociology. In contrast, explorations of concept creep emphasize the history of ideas and the psychological dimensions of conceptual change, as these are reflected in academic and public discourse and in individual minds, consistent with its origins within psychology. The theory of concept creep recognizes that shifting concepts of mental illness and mental health are closely linked to ambient macro-level cultural and societal changes but focuses its attention to the former. Ultimately, concept creep’s focus is on the dynamics of word meanings as effects of deeper cultural and societal shifts and as contributors to cultural, political, and psychological changes. In essence, concept creep is one account of the shifting understandings of distress and abnormality that underpin the broad, socially manifested phenomenon of psychiatrization.

If psychiatrization is a broader notion than concept creep in exploring the societal and institutional dimensions of the rise of psychiatric discourse, it is a narrower one in another way. Concept creep treats the expansion of psychiatric concepts as simply one example of a wider array of inflating concepts of harm. According to the theory of concept creep, concepts in the psychiatric domain such as ‘mental disorder’, ‘trauma’, and specific diagnostic entities have broadened their meanings over time, but so have ‘abuse’, ‘bullying’, ‘empathy’, ‘harassment’, ‘hate’, ‘prejudice’, ‘violence’ and many other harm-related concepts whose main field of relevance is outside or at most adjacent to that domain. Within psychology, for example, many of these concepts–which tend to involve interpersonal maltreatment rather than forms of suffering or of being harmed–are associated with developmental or social psychology rather than clinical psychology. In this respect, the semantic inflation of psychiatric concepts is one among several key domains in which concept creep takes place rather than its primary focus.

## Implications of Concept Creep for Psychiatrization

We have argued that concept creep and psychiatrization are closely aligned notions whose distinctive emphases and levels of analysis are complementary. We firmly believe that the two emerging traditions of research and theory will be mutually informative. In that spirit, we propose six implications or clarifications that our work on concept creep might offer the study of psychiatrization.

### Horizontal and Vertical Creep

The theory of concept creep distinguishes two forms of semantic expansion. Concepts may extend downward (vertical creep) to encompass less severe phenomena, and outward (horizontal creep) to include different kinds of phenomena. In the psychiatric domain, vertical creep corresponds to the relaxation of diagnostic criteria or the creation of new diagnostic entities that represent milder variants of already recognized conditions. Horizontal creep, in contrast, corresponds to the creation of qualitatively new entities, generally by colonizing new pathological territory (e.g., the addition of disorders of sleep, eating, or childhood during DSM’s evolution).

These two kinds of expansion both pathologize new forms of behavior and experience, but they have not been distinguished consistently in previous research on psychiatrization or diagnostic inflation. There is some evidence that they may have occurred to differing degrees and have different implications. For example, Fabiano and Haslam (2020) meta-analysis found no general trend for vertical creep to occur between DSM-III in 1980 and DSM-5 in 2013, despite the frequency and intensity of critiques of diagnostic expansion in this period. Although some diagnoses have unquestionably crept vertically, the horizontal expansion of psychiatry’s diagnostic reach, such as the ongoing invention of entirely new kinds of disorder, may have been more critical. Differentiating these two kinds of expansion, and their potentially different drivers and implications, could refine our understanding of psychiatrization.

The distinction between vertical and horizontal concept creep can also help to align explorations of psychiatrization with prior work on medicalization and over-diagnosis. Important work by Hofmann (2016) disentangles these frequently confused concepts, proposing that medicalization involves the extension of the medical domain into previously non-medical phenomena, whereas over-diagnosis extends existing biomedical conditions by diagnosing them when they are unlikely to cause significant suffering or impairment. On this account, medicalization is akin to horizontal concept creep whereas over-diagnosis is an example of vertical creep. Framing medicalization and over-diagnosis in this way allows them to be seen as instances of more general processes of conceptual change, analogous to shifts documented in other harm-related concepts such as prejudice and bullying (Haslam, 2016). Psychiatrization clearly involves both medicalization and over-diagnosis, and these may represent distinct dimensions of expansion in many conceptual domains.

### The Role of Harm

From the standpoint of concept creep theory, the expansion of psychiatric concepts such as mental illness and trauma is simply an instance of a generalized expansion of harm-related concepts. Research lends support to this claim, finding that disparate harm-related concepts all show the same inflationary trend, and that holding inclusive concepts of mental illness correlates with holding other inclusive harm-related concepts. The finding that the prominence of harm discourse has risen steeply–in absolute terms and relative to other moral discourses–in concert with the historical expansion of these concepts also gives credence to the centrality of harm to concept creep.

It may be instructive for those who study psychiatrization to consider it as a trend that runs in parallel to the inflation of other harm-related concepts, and that may have some shared causes and consequences. To what extent, for example, does the expansion of psychiatric concepts and categories reflect the same dynamic that drives the expansion of concepts of prejudice, abuse, bullying, and so on? Might the broadening of the psychiatric domain be linked to the rising cultural preoccupation with and sensitivity to harm that concept creep theory proposes? Does the growing interest in mental health and illness consider it primarily in terms of suffering and impairment that demands care, harm’s counterpart? Diagnostic expansion, for example, can be traced to professional bodies, lobby groups, and industrial interests, but might it also be associated with a more general cultural shift toward the acknowledgment and amplification of harm? Such an investigation might contribute to a fuller understanding of psychiatrization.

That investigation might also help to resolve a puzzling inconsistency in the political alignment of concept creep. Harm-based morality is endorsed more by people on the political left and American liberals hold relatively broader harm concepts (McGrath and Haslam, 2020). Typically, liberals are also more positively disposed than conservatives to the expansion of such harm concepts as prejudice, bullying, abuse, hate, and violence (Graham et al., 2011). Breaking this pattern, however, much of the critique of diagnostic expansion and psychiatrization or medicalization has come from the Left, who represent it as a malignant trend promoted by Big Pharma or oppressive political forces. An analysis of psychiatrization that understands it in part as a rising recognition of harm, and a corresponding expansion of certain forms of care, might complicate the views of some left-leaning critics of psychiatrization by revealing another, more progressive dynamic at play.

### Benefits of Psychiatrization

The general tone of much research on psychiatrization is critical. Undoubtedly the risks of over-diagnosis, over-treatment, and resource misallocation are serious, and there are cultural costs associated with the adoption of a psychiatric idiom to understand everyday unhappiness. However, psychiatrization is also sure to have benefits. Concept creep theory has consistently maintained that shifting concepts of harm are likely to have mixed blessings: it draws attention to neglected harms but also inflates minor harms in problematic ways. Concept creep research (e.g., McGrath et al., 2019) has shown that holding broad concepts of harm, such as expansive definitions of mental illness, is associated with a mix of desirable and undesirable attributes and outcomes. Without making the naïve assumption that the benefits and costs of psychiatrization are equal, viewing it through the lens of concept creep may clarify its ambivalent character. Beeker et al. (2021) recognize this ambivalence in their overview of the topic, and concept creep provides a way to think through its benefits and costs.

The work of Tse and Haslam (2021) is a case in point, providing a detailed account of how concept creep might have specific benefits. They demonstrated that holding favorable attitudes to mental health help-seeking is associated with more inclusive concepts of mental disorder, and that cultural differences in the inclusiveness of these concepts are associated with differences in help-seeking. This research implies that in under-served populations, promoting broadened concepts of psychiatry’s domain might have positive effects. Other work inspired by concept creep presents an equally detailed analysis of the costs of psychiatrization. Jones and McNally, (2021) found that people experimentally induced to hold a more expansive concept of trauma were more likely to experience lasting psychological effects after being exposed to a disturbing video. This work illustrates how the dissemination of broadened psychiatric concepts may foster vulnerability in the general public. The concept creep framework, which views any broadening of harm-related concepts as a potential acknowledgement of previously neglected harm and an opportunity for beneficial care, also recognizes it as a potential source of vulnerability, fragility, and unwarranted intervention.

### Variation

Although a large proportion of concept creep research has examined it as a historical phenomenon, another significant focus has been on variability among individuals and groups in the adoption of broad concepts of harm. Our studies have shown that people who tend to have broader concepts–and might therefore be viewed as at the vanguard of harm inflation–tend to be politically liberal, female, high in empathy and sensitive to injustice, likely to endorse an individualist harm-based morality, and, in some cases young and liable to a sense of personal vulnerability. This work has not systematically examined variability in the breadth of psychiatric concepts–although these were included among others–but such an investigation might be pertinent to the study of psychiatrization. Although psychiatrization is primarily understood as a historical trend, it is unlikely to have unfolded in uniform ways by different groups or to have been universally accepted or rejected by them. It may be instructive to learn which groups of people–based on nationality, culture, race, gender, ideology, personality, age, and so on–are most likely to embrace or resist the rise of psychiatric discourse and the expansion of diagnostic categories. These differences, the pursuit of which has been central to concept creep research, may have implications for the future of psychiatrization.

### Professional Versus Lay Concepts

The theory of concept creep proposes that broadened concepts of harm often originate in the academy and the professions, and then diffuse through the culture at large. In the psychiatric domain, for example, expansive concepts of mental illness may originate in psychiatry’s diagnostic manuals and then disseminate into the wider public through education, the media, and encounters with professionals. Psychiatrization is understood to involve professionals and specialist interests as well as the citizenry, and to include the institutional practices of mental health professionals and their associated institutions as well as the everyday language use of laypeople, so the nature of the relationship between these ‘top level’ and ‘bottom level’ actors (Beeker et al., 2021) is important for understanding it. By recognizing a combination of top-down and bottom-up processes that jointly produce psychiatrization, Beeker and others acknowledge that the links between these levels are important to grasp.

One lesson from concept creep is that such links are likely to be complex and a simple dissemination process cannot account for it. (Haslam et al., 2016) examined changes in the relative frequency and semantic breadth of a collection of harm-related psychological concepts such as ‘trauma’ over the past 40 years in two text corpora, one professional (the abstracts of close to 800,000 psychology articles) and the other representing general cultural content (e.g., magazine and news articles, transcripts of TV programs, fiction). If the popularity or semantic inflation of the concepts disseminated from professional discourse into the wider culture, then lagged relationships should be evident in the frequency and breadth of the concepts between the two corpora, with shifts in the psychology corpus predating shifts in the general corpus. Such relationships were very scarce. Instead, it appeared that the popularity and breadth of the concepts varied within the two corpora in ways that were weakly couple, if at all. Understanding how top-down psychiatrization processes operate should be a priority for future research, but our work suggests these processes may be subtle.

A related observation we would make is that some of the discourse that may be driving psychiatrization, and the concept creep of ‘mental illness’ that underpins it, may not emanate from psychiatry at all but from adjacent studies of well-being within psychology and cognate fields. We have observed a growing tendency in these disciplines to conflate ‘well-being’ with ‘mental health’ that is likely to produce a tendency to pathologize what Freud called “common unhappiness”. Whereas once ‘mental health’ was understood primarily as the absence of mental illness and ‘well-being’ as the presence of desirable emotional states and satisfaction with life, when the two concepts are conflated ‘mental health’ is increasingly represented as a positive state of health beyond the absence of symptoms. However, if ‘mental health’ becomes a salutary state that is close to synonymous with ‘well-being’, then states of low well-being risk being seen as mental illness.

Systematic research on word associations supports the view that in the general public ‘mental health’ has a dual aspect, representing a positive state of well-being but also retaining strong associations with pathology. Using (De Deyne et al., 2019) massive ‘small word of words’ database, which contains associations for more than 15,000 words made by over 100,000 people, we have examined the mental associations of ‘well-being’ and ‘mental health’. For each concept, directed weighted ego-graphs were extracted based on: 1) forward associations (e.g., responses to ‘well-being’ or ‘mental health’), 2) backward associations (cue words that elicited ‘well-being’ or ‘mental health’ as a response), and 3) all edges between words in the ego-graph. For simplicity, infrequent responses (i.e., associative strength <0.04 on a scale from 0 to 1) were removed. To visualize the relation between words, hierarchal walktrap clustering was applied using the R igraph package (Csardi and Nepusz, 2006).

**FIGURE 1:**
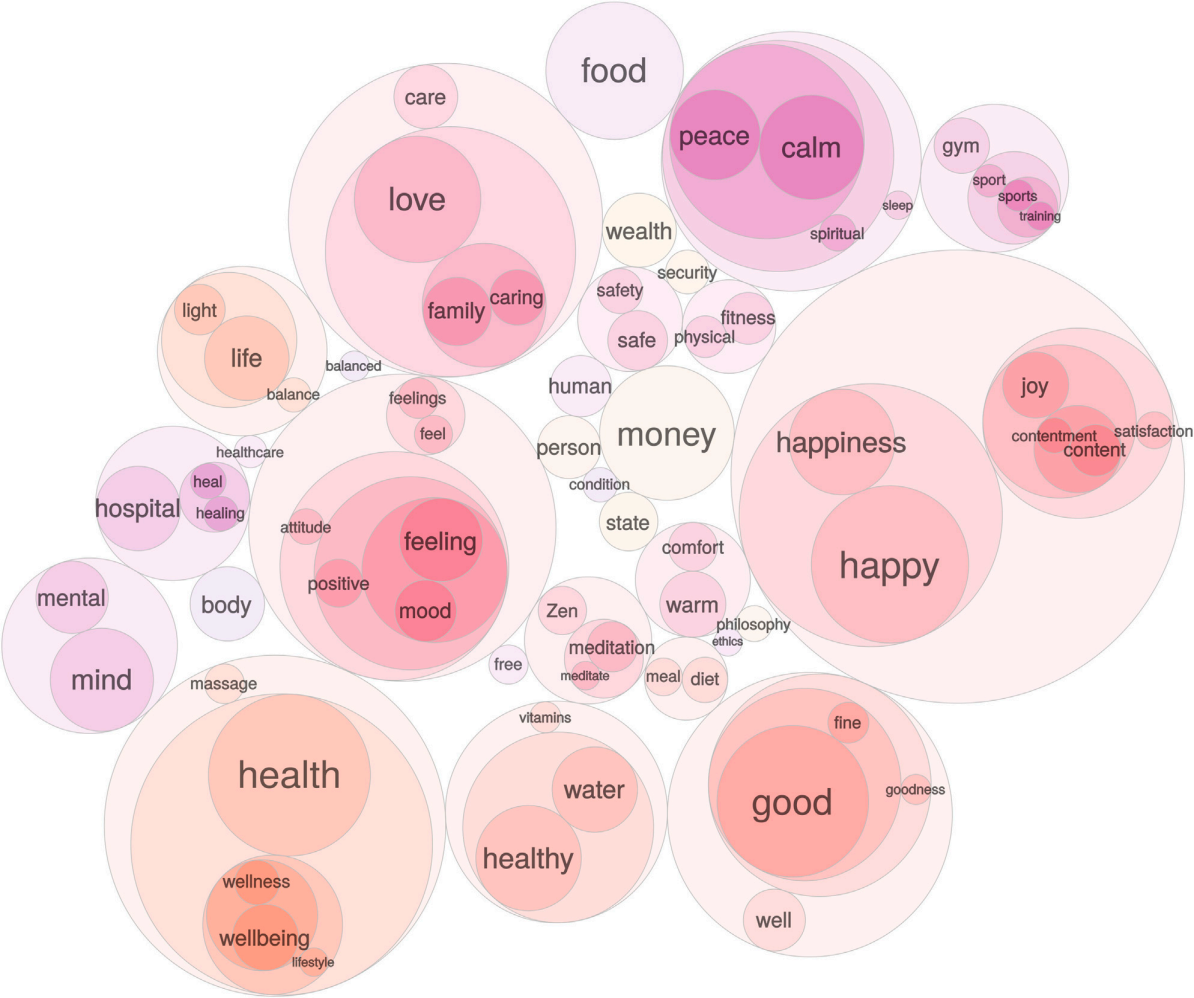
Word association egograph for “well-being”. Colors indicate distinct clusters of meaning extracted using walktrap clustering. Cluster size indicates the prevalence of association responses measured as response in-strength (i.e., the sum of weighted incoming edges).

**FIGURE 2:**
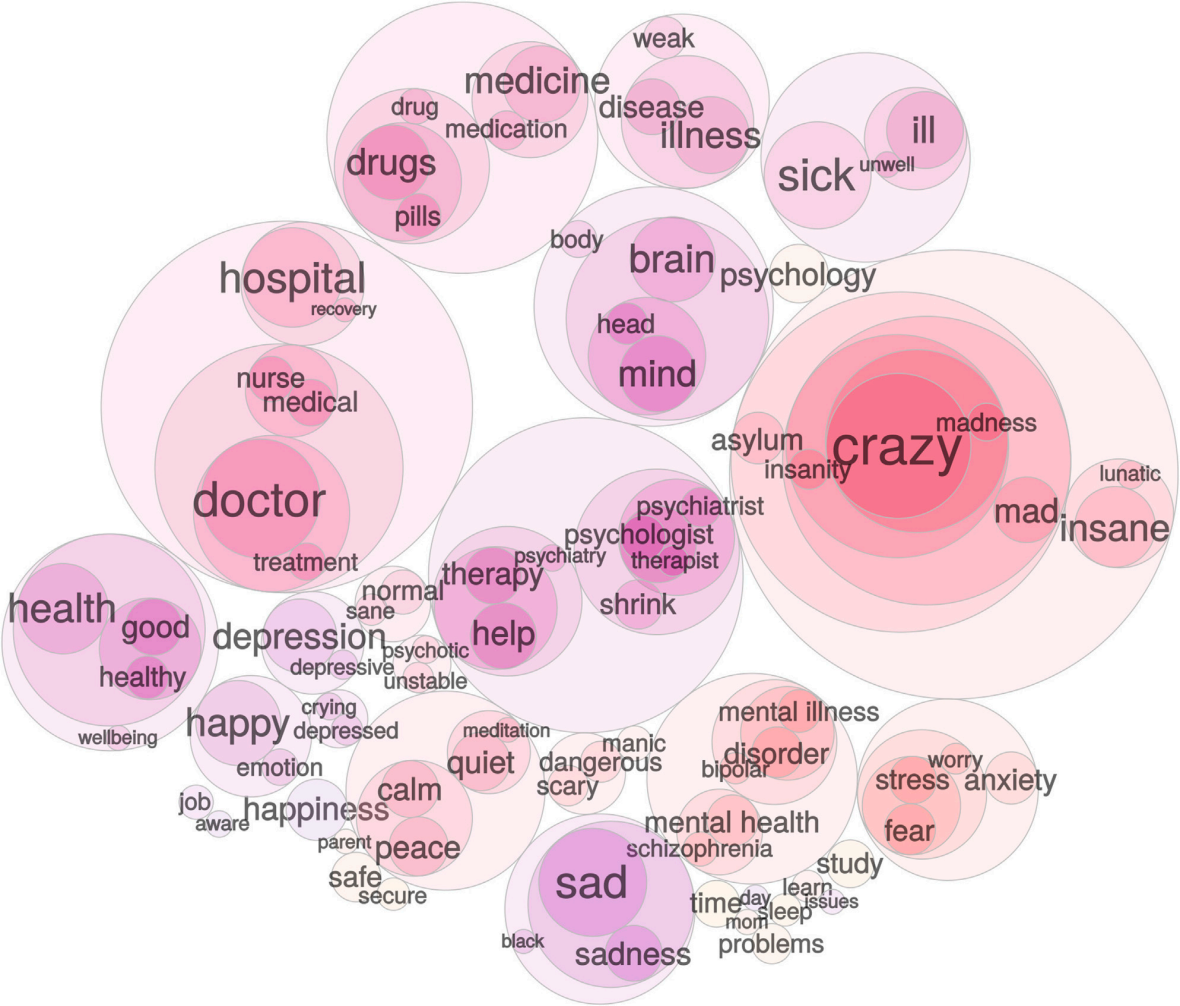
Word association egograph for the word “mental health”. Colors indicate distinct clusters of meaning extracted using walktrap clustering. Cluster size indicates the prevalence of association responses measured as response in-strength (i.e., the sum of weighted incoming edges).

Figures 1, 2 present visualizations of the two terms’ associations. ‘Well-being’ (Figure 1) is associated with a range of desirable states and their causes. However, ‘mental health’ (Figure 2), though ostensibly a positive concept and a near-synonym of ‘well-being’, is primarily associated with words referring to mental illness and its treatment, as well as diagnostic labels, stigmatizing terms, and unpleasant emotional states. ‘Mental health’ carries with it the shadow of pathology, and as it is increasingly employed as a substitute for or fellow traveller with ‘well-being’ it is likely to extend psychiatrization into the domain of suboptimal well-being. By implication, psychiatrization must be studied as a top-down process that emanates from a wider range of sources than psychiatry and the mental health industry narrowly conceived.

## Conclusion

The concept of psychiatrization is a powerful one that has the potential to integrate several lines of theory and research on the causes and effects of the rising prominence of psychiatric concepts and practices. We applaud its integrative possibilities and the openness of its proponents to transdisciplinary research efforts. Research and theory on concept creep have a role to play in enhancing our understanding of psychiatrization and in framing new approaches to studying it.
